# Monthly Summary

**Published:** 1869-12

**Authors:** 


### MONTHLY SUMMARY.

*Tetanus.*—Dr. Dunlap was called on the night of March 20th, to see Miss S. Found her with jaw firmly set; one of the attendants had taken the precaution to place a stick between her teeth, which left room to administer remedies. She had been in that condition about six hours. I learned that about two weeks previous to that time she had singed her foot on the under side, and had so neglected the wound that it was dirty, swollen, suppurating and very offensive. I immediately ordered comp. syrup morphia, spirits vini Gallici aa f℥ij. Teaspoonful every ten minutes. Also ordered chloroform by inhalation. As soon as the chloroform took effect, the muscles relaxed, the stick fell from her mouth, and she seemed in every way comfortable. In about one hour she rallied from the effects of the chloroform and talked. The mixture of syrup morphia was continued in teaspoonful doses every half hour for three hours, until she quietly fell asleep. In the meantime the wound had been cleansed, and poulticed. When I called again next day I found her walking about and feeling quite well.—*Med. & Surg. Reporter.*

---

*Aluminum.*—The scientific editor of the *Independent* says: In his *Journal of Applied Chemistry*, Professor Joy says that a few years ago a pound of aluminum could not have been purchased for $200, and even at that price there were few manufacturers hardy enough to take the order. At the present time it can be readily manufactured for seventy-five cents, if not for fifty cents a pound; and the probabilities are that we shall soon be able to obtain it for a quarter of a dollar. Its principal use thus far is in the manufacture of aluminum bronze, or alloy of copper with ten per cent. of aluminum, and which possesses remarkable strength, tenacity, and elasticity.

Aluminum will prove a very important and useful metal: useful in the manufacture of surgical instruments and appliances etc., and we are glad that it has at last reached the inevitable point of a material reduction in price.

---

*Chloral—A New Anæsthetic.*—Monsieur Liebreich has presented a memoir to the Academie des Sciences, which contains some interesting details concerning a new anæsthetic he has just discovered. An important difference between this new chemical compound, which he calls "chloral," and all other substances used for the purpose of producing insensibility, is, that it is administered by absorption instead of inhalation, and this enables the dose applied to be measured with greater accuracy. On passing into the system it becomes decomposed into formiate of potassium and chloroform, and produces more perfect insensibility than either ordinary chloroform or ether. Its use is said to be unattended by any danger. In a very painful and difficult operation lately performed on a woman, M. Liebreich applied chloral with perfect success, the patient being kept under its influence for over two hours.—*Med. & Surg. Reporter.*

*Why is the Production of Pus a Cause of Exhaustion?*—Every one knows that when a patient is suffering from a suppurating wound or abscess, his system becomes rapidly exhausted. What is the cause of this? Is there anything in the material of pus to explain it?

Pus, as seen under the microscope, consists of two parts, a fluid portion called the *liquor puris*, and certain histological elements, called *cytoid or pus corpuscles*. The former is a clear, colorless or slightly yellowish fluid with a feeble alkaline reaction. The latter are organized materials, and consists of a cell membrane, with a nucleus adhering to it, and viscid hyaline contents.

In these organized materials lies the secret of systemic waste in large suppurating wounds. Their production necessitates a large expenditure of vital force—quite as large as does the embryo material for the building up of true tissue. It is not the mere loss of material, but it is this outlay of vital force that causes exhaustion to the suppurating patient.

For this explanation of this hitherto unexplained difficulty the scientific world is indebted to Mr. Paget, surgeon to St. Bartholomew's Hospital, London; and he, as he modestly intimates, to his house-surgeon, Mr. Butcher.—*Am. Eclectic Med. Review.*

---

*Death from Bichloride of Methylene.*—The first recorded death (as far as we are aware) from the inhalation of bichloride of methylene occurred this week in Charing-Cross Hospital. The patient, who had been greatly reduced by malignant disease of the jaw, was about to be operated on by Mr. Canton. The anæsthetic agent was being administered by Mr. Peter Marshall, who has had great experince in its use, and only a very small quantity had been given when the fatal collapse occurred.

---

*A Young Tobacco Chewer.*—In the *Medical & Surgical Reporter* a physician of Portage county, Ohio, relates a case within his knowledge where a boy, now some fifteen years old, has used tobacco since the age of five months. When five months old, being a nervous and fretful child, a plug of tobacco was placed in his mouth and produced a soothing effect. The remedy was often used during infancy, and through the teething period, and before the child could talk plainly it was a confirmed tobacco chewer.

*Tobacco—The Quantity Consumed and its Effect on Youth.*—Few, perhaps, are aware of the extent to which the weed is used both in Europe and America. And its consumption seems to be on the increase. The quantity, in ounces, per head, which is consumed in the United States and Europe is estimated as follows:—Belgium, 150 to 160; Holland, 130; Denmark, 120; United States, 120; Austria, 110; Norway, 100; France, 80; Spain, 70 to 80; Sweden, 70; Great Britian, 60 to 70; Portugal, 50 to 60; Russia, 40; Sardinia, 40; Tuscany, 30 to 40.

In the year 1852, the number of cigars consumed in the city of Paris alone was 200,000,000; in the year 1867, the number increased to 761,625,000.

It is said that a very marked degeneracy of Parisian physique has been noticeable in late years, and that one of the causes of this degeneracy is undoubtedly the excessive use of tobacco.

A writer in the *British Medical Review* says:

Dr. Decaisne, in the course of investigations on the influence of tobacco on circulation, has been struck with the large number of boys, aged from 9 to 15 years, who smoke; and has been led to inquire into the connection of this habit with the impairment of the general health. He has observed 38 boys, aged from 9 to 15, who smoked more or less. Of these, distinct symptoms were presented in 27. In 22 there were various disorders of the circulation—*bruit de souffle* in the neck, palpitation, disorders of digestion, slowness of intellect, and a more or less marked taste for strong drinks. In 2 the pulse was intermittent. In 8 there was found more or less diminution of the red corpuscles. In 12 there were rather frequent epistaxes: 10 had disturbed sleep, and 4 had slight ulcerations of the mucous membrane of the mouth, which disappeared on ceasing from the use of tobacco for some days. In children who were very well nourished, the disorder was, in general, less marked. As to the ages, 8 of the boys were 9 to 12 years old: 19, from 12 to 15. The duration of the habit of smoking was in 11 from 6 months to a year; and in 16 more than two years. The ordinary treatment of anæmia in general produced no effect as long as the smoking was continued; but when this was desisted from, health was soon perfectly restored, if there was no organic disease.—*American Eclectic Med. Review.*

